# Electrothermal Synthesis of Cell-Imprinted Polymer Coatings on Metallic Microwires for Bacterial Capture

**DOI:** 10.3390/s26175324

**Published:** 2026-08-22

**Authors:** Alireza Zabihihesari, Arezoo Khalili, Pouya Rezai

**Affiliations:** 1 Department of Mechanical Engineering, York University, Toronto, ON M3J 1P3, Canada; alireza.zabihihesari@dal.ca (A.Z.); arezookhalili1050@gmail.com (A.K.); 2 Department of Electrical and Computer Engineering, Dalhousie University, Halifax, NS B3H 4R2, Canada; 3 Department of Mechanical Engineering, George Mason University, Fairfax, VA 22030, USA

**Keywords:** imprinted polymers, electrothermal polymerization, joule heating, bacteria capture

## Abstract

**Highlights:**

**What are the main findings?**

- A localized electrothermal polymerization method was developed to fabricate cell-imprinted polymer coatings directly on metallic microwires.
- Cell-imprinted polymer-coated microwires achieved significantly higher *Salmonella* capture efficiency (~70%) than bare and non-imprinted polymer-coated controls.

**What are the implications of the main findings?**

- Localized Joule heating enables spatially controlled fabrication of cell-imprinted polymer coatings on non-planar metallic microwires.
- The resulting CIP-coated microwires demonstrate proof-of-concept bacterial capture with improved performance relative to bare and non-imprinted controls.

**Abstract:**

This study presents an electrothermal coating approach for synthesizing cell-imprinted polymers (CIPs) on metallic microwires through localized resistive heating-induced polymerization. Imprinted polymers (IPs) are robust, cost-effective synthetic affinity materials widely used in sensing applications. However, conventional fabrication methods, including bulk and suspension polymerization, often lack spatial control, producing non-specific polymerization, heterogeneous coatings, and reduced sensor reproducibility. Electrochemical polymerization provides improved spatial control but requires specialized instrumentation and restricts monomer selection. Here, applying direct current (DC) to metallic microwires immersed in a prepolymer solution generated localized Joule heating, enabling controlled in situ polymerization and uniform coatings while minimizing undesired bulk polymerization. By optimizing the applied current and polymerization time, CIP coatings with tunable thicknesses were fabricated on gold-coated microwires. Under optimized conditions, ~6 µm thick coatings were imprinted using *Salmonella* templates. Scanning electron microscopy revealed bacteria-shaped cavities consistent with template removal and the formation of imprinted cavities. Rebinding experiments demonstrated enhanced bacterial capture, with CIP-coated microwires achieving ~70% capture efficiency, compared to 22% for bare microwires and 33% for non-imprinted polymer (NIP) controls. These results support the effectiveness of the proposed method for localized polymerization and demonstrate the enhanced capture of the template species by CIP-coated microwires relative to bare microwires and NIP-coated controls.

## 1. Introduction

The rapid, reliable, and low-cost detection of bacterial and viral pathogens is essential for biomedical diagnostics, food safety, and environmental monitoring [1]. In clinical settings, timely identification and the isolation of bacterial pathogens are crucial for antimicrobial susceptibility testing (AST), enabling appropriate antibiotic selection and improved patient outcomes. Conventional phenotypic AST methods, such as broth microdilution, disk diffusion, gradient diffusion, and automated culture-based systems, remain the clinical gold standard [2]. However, these methods typically require bacterial isolation and prolonged incubation, resulting in turnaround times of 24–72 h or longer. Molecular methods, based on polymerase chain reaction (PCR) and immunoassays such as enzyme-linked immunosorbent assays (ELISA), are faster but are often costly, labor-intensive, or dependent on specialized instrumentation and trained personnel [3]. Recent advances have combined microfluidics, imaging, and machine-learning-assisted analyses to enable rapid bacterial detection and AST across clinically important infections, including tuberculosis, sepsis, urinary tract infections, and heteroresistant bacterial populations [4,5,6,7]. Despite these advancements, most existing technologies remain laboratory-based and are not readily suited for point-of-need applications in biomedical and environmental monitoring, highlighting the need for rapid, inexpensive, and portable platforms for selective bacteria capture and detection. To address these limitations, biosensors integrating a biorecognition element with a signal transducer have emerged as promising alternatives for rapid pathogen detection [8,9]. Among these, electrochemical biosensors are particularly attractive because of their high sensitivity, specificity, portability, low cost, and short assay times, enabling real-time and point-of-care analysis [10,11,12,13].

Despite their widespread use, natural receptors such as antibodies and enzymes have several limitations, including poor stability under harsh environmental conditions, high production cost, limited shelf life, dependence on animal-derived sources, and stringent storage conditions to prevent degradation and denaturation [14]. Consequently, significant research efforts have focused on developing synthetic recognition materials that mimic the selectivity of biological receptors while offering enhanced robustness and long-term stability [15]. Imprinted polymers (IPs) have emerged as durable, selective, and cost-effective synthetic affinity materials for recognizing a broad range of analytes, including molecules (MIPs) [16,17,18,19,20], ions (IIPs) [21,22,23,24], and cells (CIPs) [25,26,27,28,29].

IP synthesis involves copolymerizing functional monomers and cross-linkers in the presence of the target analyte, known as the template. Following template removal, complementary recognition cavities remain within the polymer matrix, retaining the morphological and chemical characteristics of the target and enabling selective rebinding for analyte capture and detection [30].

Depending on the polymerization technique and application, IPs can be synthesized as bulk monoliths [31], micro/nanoparticles [32], membranes [33,34], or thin films [35]. For sensing applications, thin IP coatings are commonly deposited onto transducers such as electrochemical electrodes, fluorescent particles, optical substrates, and quartz crystal microbalance (QCM) surfaces [36,37,38].

Various techniques have been developed to fabricate thin IP coatings on both planar transducers, including screen-printed electrodes (SPEs) [39,40,41,42,43] and non-planar structures such as microspheres and microwires [44,45,46]. Conventional techniques for polymerizing IP coatings include bulk [17], suspension [47], precipitation [48], and electrochemical [49] polymerization. These approaches typically require prior surface functionalization of the transducer to promote covalent attachment between the polymer coating and the substrate [50]. Polymerization is subsequently initiated thermally, photochemically, or electrochemically using suitable initiators [39,50].

In conventional thermal and photopolymerization methods, the entire prepolymer solution is typically exposed uniformly to heat (e.g., oven-based thermal polymerization) or light irradiation without precise spatial control. As a result, polymerization may occur beyond the transducer surface, leading to non-specific polymer formation, heterogeneous coating thicknesses, and reduced reproducibility. Furthermore, bulk polymerization often produces highly cross-linked rigid monolithic or glassy structures that require extensive post-processing, including grinding and sieving, to obtain usable particles or coatings [51]. Electrochemical polymerization provides improved spatial control because polymer growth is confined to the electrode surface. However, this technique requires specialized instrumentation and restricts the selection of monomers and cross-linkers to electrochemically compatible systems capable of undergoing controlled redox reactions without significant degradation, side reactions, or electrode fouling [52].

Although conventional approaches have enabled the fabrication of thickness-controlled IP films on planar substrates such as SPEs [53,54,55], extending these methods to non-planar transducers remains challenging [29,44,45]. Moreover, while extensive efforts have focused on molecularly and ion-imprinted systems, comparatively less attention has been directed toward the fabrication of transducer-integrated cell-imprinted polymers for larger biological targets such as bacteria and viruses [56].

Here, we present an electrothermal coating strategy for the localized synthesis of cell-imprinted polymers (CIPs) on metallic microwires. By applying direct current (DC) through the microwires, localized Joule heating is generated, enabling the controlled thermal polymerization of the prepolymer solution in the immediate vicinity of the microwire surface. Using this approach, thickness-controlled *Salmonella*-imprinted polymer coatings containing distinct bacteria-shaped cavities were successfully fabricated on gold-coated microwires. The resulting CIP-coated microwires demonstrated substantially higher bacterial capture efficiency than bare and non-imprinted polymer-coated controls. The present study establishes the feasibility of localized electrothermal polymerization for fabricating CIP coatings on metallic microwires and enhancing the capture of the template bacterial species. Integration into operational sensing or microfluidic platforms requires further investigation.

## 2. Materials and Methods

### 2.1. Materials

Gold-coated microwires (128 µm diameter, Product ID: 50364, MWS, Oxnard, CA, USA) were used as substrates for CIP coating. For the surface treatment of microwires and preparation of CIP prepolymer solution, acrylamide (AAM), methacrylic acid (MAA), methyl methacrylate (MMA), N-vinylpyrrolidone (NVP), dimethyl sulfoxide (DMSO), 2,2′-azobisisobutyronitrile (AIBN), N,N′-(1,2-dihydroxyethylene) bisacrylamide (DHEBA), sulfuric acid, ethanol, hydrogen peroxide, 11-mercaptoundecanoic acid (MUA), N- hydroxysuccinimide (NHS), N-(3-Dimethylaminopropyl)-N′-ethylcarbodiimide hydrochloride (EDC), 2,2′-azobis (2-amidinopropane) hydrochloride (ABAH), hydrochloric acid (HCl) and Rhodamine 110 chloride fluorescent dye (497 nm blue excitation and 520 nm green emission) were purchased from Sigma-Aldrich (St. Louis, MO, USA).

*Salmonella typhimurium* (ATCC 14028) strain obtained from Carolina^®^ Biological Supply Company (Burlington, NC, USA) was used as both the template organism for CIP fabrication and the target analyte for bacterial rebinding experiments.

### 2.2. Surface Functionalization of Gold-Coated Microwires

Prior to functionalization, the microwires were cut into 4 cm long segments and washed with 4 mL of freshly prepared piranha solution (1 mL H_2_O_2_ + 3 mL H_2_SO_4_) for 3 min, followed by sequential rinsing with deionized (DI) water and absolute ethanol (three times each). The cleaned microwires were then dried under nitrogen gas.

To form a self-assembled thiol monolayer on the gold surface, the microwires were immersed overnight at room temperature in 10 mL of a 1 mM ethanolic solution of MUA. Following incubation, the microwires were rinsed thoroughly with ethanol and DI water to remove unbound thiols and subsequently dried under nitrogen.

**Figure 1:**
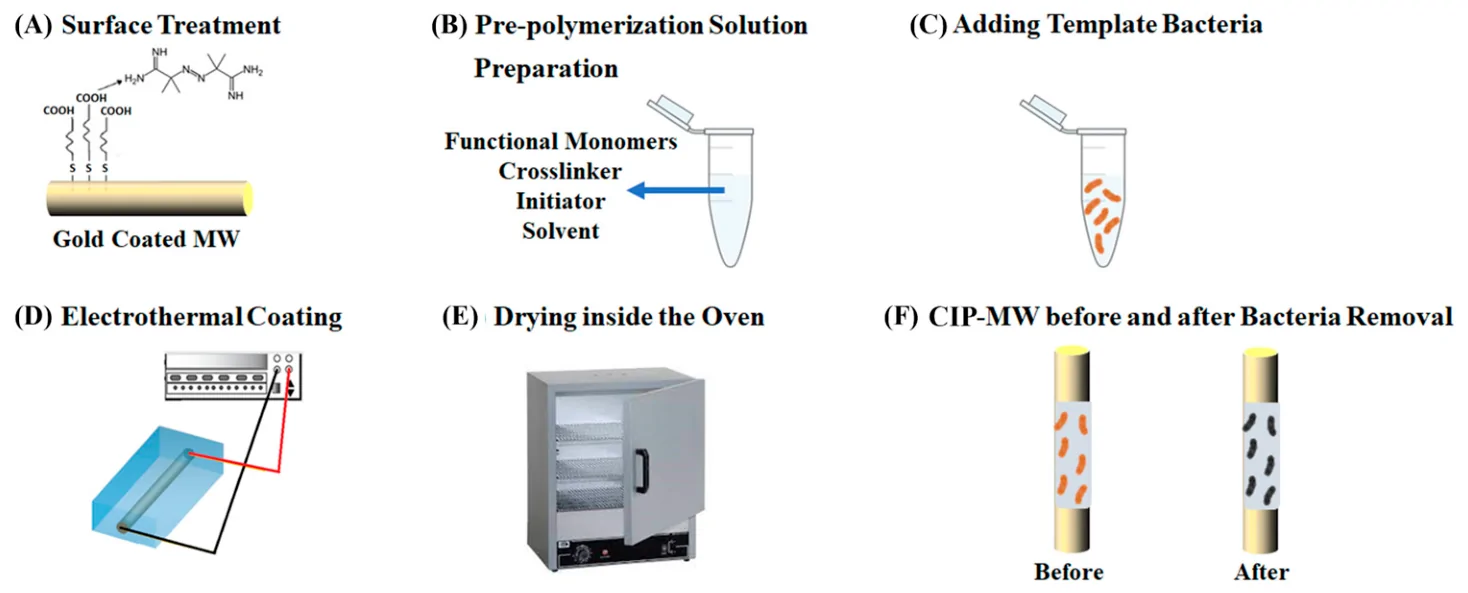
Schematic illustration of the fabrication process for cell-imprinted polymer-coated microwires (CIP-MW). (**A**) Surface functionalization of gold-coated microwires; (**B**) preparation of the CIP prepolymer solution; (**C**) incorporation of *Salmonella* template bacteria into the prepolymer solution; (**D**) electrothermal coating of microwires through localized Joule heating-induced polymerization; (**E**) completion of polymerization in an oven; and (**F**) formation of CIP coatings on microwires before and after removal of the template bacteria.

Surface carboxyl groups were also activated by placing microwires in a 5 mL aqueous solution of NHS (0.05 M) and EDC (0.2 M) for 60 min. The microwires were then transferred into 10 mL ABAH (200 mM) aqueous solution and incubated at room temperature for an additional 180 min. Finally, the functionalized microwires were dried under a nitrogen stream prior to exposure to the prepolymer solution (Figure 1A).

### 2.3. Preparation of Prepolymer Solution

The prepolymer formulation was adapted from the virus-imprinted polymer composite formulation previously reported by Sukjee et al. [57]. Functional monomers (60 mg AAM, 24 μL MAA, 48 μL MMA and 48 μL NVP) were mixed in 600 μL DMSO. Then, 94 mg DHEBA and 3 mg AIBN were added as cross-linking agent and initiator, respectively (Figure 1B). To enable fluorescent visualization of the resulting polymer coatings, Rhodamine 110 chloride fluorescent dye (100 μL, 1 mM in DI water) was added into the prepolymer solution.

### 2.4. Bacteria Culturing

Fresh single-colony isolates of *Salmonella typhimurium* (ATCC 14028) were cultured in Luria–Bertani (LB) broth consisting of 5 g NaCl, 10 g Bacto™ tryptone, and 5 g Bacto™ yeast extract dissolved in 1 L of distilled water. The bacterial cultures were incubated at 37 °C with continuous shaking at 150 rpm for 24 h. Following incubation, bacterial suspensions with concentrations of approximately 10^9^ CFU/mL were used for the fabrication of CIP-microwires.

For rebinding experiments, the bacterial cultures were serially diluted in LB broth to obtain the desired concentration of approximately 10^4^ CFU/mL. Standard plate culturing and colony-counting methods were used to determine bacterial concentrations when required [58,59].

All experiments involving bacterial culturing were conducted in a biosafety level 2 (BSL-2) laboratory in accordance with York University biosafety regulations and permit PR-02-19.

### 2.5. Preparation of Microwires Coated with Non-Imprinted Polymers

**Figure 2:**
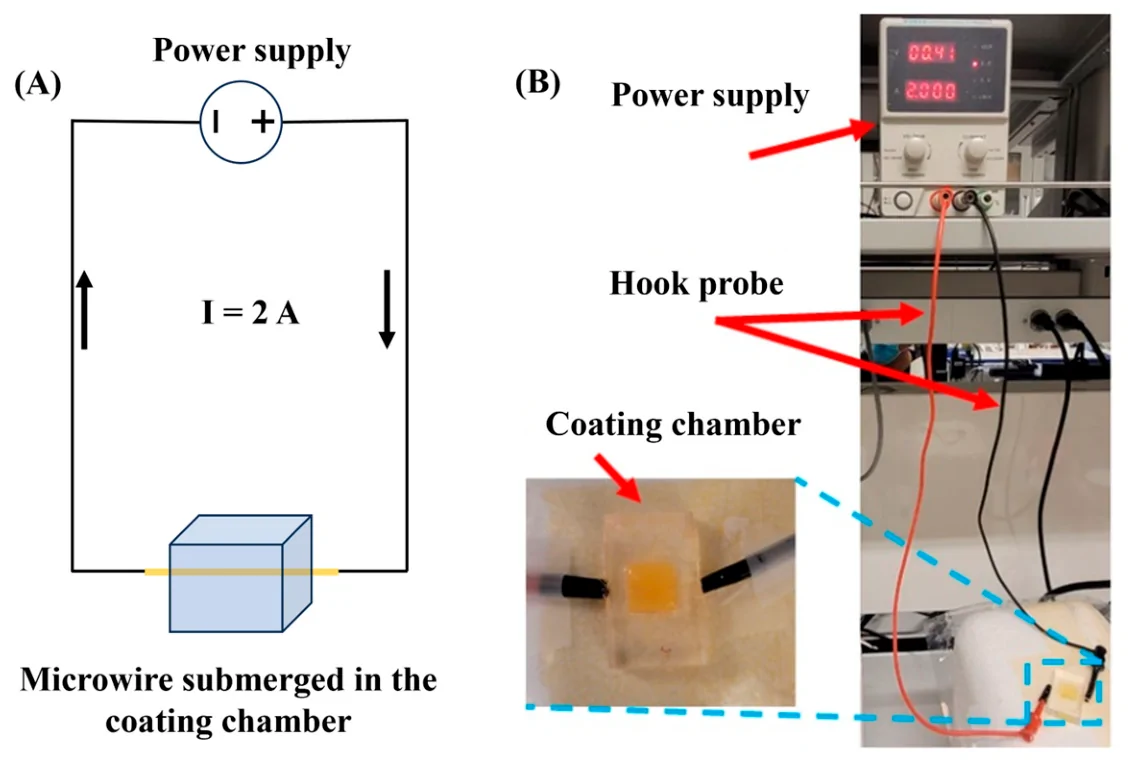
Experimental setup. (**A**) Schematic illustration, and (**B**) photograph of the experimental setup, including the power supply, electrical connections, and polymerization chamber.

Microwires coated with non-imprinted polymers (NIPs) were fabricated using the electrothermal polymerization approach illustrated in Figure 1D. The experimental setup consisted of a DC power supply (KD3005D, KORAD, Dongguan, China), two hook probes for electrical connection to the microwires, and a custom polymerization chamber containing grooves for positioning the microwires during coating (Figure 2).

The polymerization chamber was fabricated from polydimethylsiloxane (PDMS) using standard soft-lithography techniques. Briefly, PDMS prepolymer and curing agent were mixed at a 10:1 ratio, poured onto a 3D-printed master mold, degassed under vacuum to remove trapped air bubbles, and thermally cured. Following curing, the patterned PDMS layer was peeled from the mold and irreversibly bonded to a glass substrate using oxygen plasma treatment to form a sealed polymerization chamber. To support and position the functionalized microwires during polymerization, shallow grooves were manually introduced on opposite sides of the PDMS chamber using a sharp blade. The grooves enabled the microwires to be suspended and aligned at the center of the chamber while maintaining mechanical stability throughout the coating process. The chamber was subsequently filled with the prepolymer solution, ensuring complete immersion of the microwire within the polymerization region prior to electrothermal polymerization.

Constant electric currents (I) of 1.5, 2.0, and 2.5 A were applied in separate experiments to generate localized Joule heating for initiating thermal polymerization and coating formation on the microwire surface. During each electrothermal exposure, the output voltage displayed by the DC power supply was recorded. The effective electrical resistance of the experimental circuit and the corresponding electrical power supplied by the source were calculated as $R=\frac{V}{I}$ and P=VI, respectively. The calculated resistance may include contributions from the microwire, electrical contacts, and connecting leads. Accordingly, the resistance and power dissipation of the microwire alone were not independently quantified. During current application, a gel-like polymer layer formed selectively around the microwire, while the surrounding prepolymer solution remained in liquid form. Following electrothermal coating, the excess prepolymer solution was removed from the chamber, and the coated microwire was carefully transferred to an air-circulated gravity convection oven (Heratherm™, Thermo Fisher Scientific, Waltham, MA, USA) and incubated at 55 °C for 12 h to complete polymerization (Figure 1E). After determining the optimal current for stable coating formation, subsequent experiments varied the electrothermal exposure time between 1 and 6 h to optimize coating uniformity and thickness.

To measure the temperature of the microwires under varying currents, we employed a sensitive temperature sensor connected to a digital multimeter (BK Precision 2706B). A secure connection between the sensor and the microwire was ensured to guarantee accurate temperature readings. An optical microscope (DMIL LED Inverted Microscope, Leica, Wetzlar, Germany) captured images of the microwires, which were then analyzed using a custom macro in ImageJ (version 1.54p) software to measure their diameter, as described in [45] and in the Supplementary Information (Note S1, Figure S1 and Table S1). To evaluate coating uniformity, a diameter-based uniformity index was calculated as

(1) $$U=\left(1-\frac{SD}{\bar{D}}\right)$$

where $\bar{D}$ and *SD* are the mean and standard deviation of microwire diameter from 100 measurements performed by ImageJ Macro along a ~2 mm length of the microwire. Values closer to one indicate greater uniformity. Also, the lengths of all visibly coated segments in fluorescent images were measured and summed. Then, longitudinal coating coverage was calculated as

(2) $$Longitudinal\;coverage\;(\%)=\frac{\sum L_{coated}}{L_{analyzed}}\times 100$$

where $\sum L_{coated}$ is the cumulative length of all coated segments within a randomly selected, ~2 mm-long region of the microwire, and $L_{analyzed}$ is the total microwire length visible within the analyzed region (Table S2). This measurement represents *longitudinal coverage* within the two-dimensional fluorescence image and does not quantify circumferential surface coverage.

The morphology of the coated microwires was further characterized using scanning electron microscopy (SEM) (Quanta 3D FEG, Thermo Fisher Scientific).

### 2.6. Preparation of CIP-Coated Microwires

To prepare CIP-coated microwires, 1 mL of bacteria suspension in LB (10^9^ CFU/mL) was centrifuged at 1200 rpm for 2 min, forming a bacterial pellet at the bottom and an LB supernatant at the top of the centrifuge tube. The supernatant was carefully removed using a micropipette and replaced with 4 mL of prepolymer solution. The bacteria were resuspended in the prepolymer solution by vortexing for 2 min to obtain a homogeneous bacterial suspension (Figure 1C). Next, 1 mL of the prepared solution was transferred into the coating chamber, where surface-functionalized microwires had already been positioned. CIP-coated microwires were polymerized using the same electrothermal procedure described for NIP-coated microwires in Section 2.5. After polymerization, the microwires were soaked in HCl (1 M) for 1 h and then transferred to DI water and incubated at 50 °C for 30 min to remove the bacterial templates from the CIP coatings, creating cavities for rebinding (Figure 1F).

### 2.7. Rebinding Assay

A 1 cm CIP-coated microwire was immersed in a 300 µL *Salmonella* solution with a concentration of ~10^4^ CFU/mL. After 30 min of incubation on a shaker at room temperature, the microwire was removed from the solution. Using plate culturing and colony counting (Supplementary Information Note S2 and Table S3), the initial bacterial concentration of the primary solution ($B_{t}$) and the residual bacterial concentration after microwire removal ($B_{r}$) were determined. The captured bacterial concentration ($B_{c}$) was then calculated using equation (Equation (3)):

(3) $$B_{c}=B_{t}-B_{r}$$

Then, the bacterial capturing efficiency was calculated as

(4) $$Bacterial\;capturing\;efficiency\;\left(\%\right)=\left(\frac{B_{t}-B_{r}}{B_{t}}\right)\times 100$$

In all experiments, the total number of colonies was counted after 20 h incubation at 37 °C. Control experiments were also conducted using bare and NIP-coated microwires. Statistical comparisons were performed using two-tailed Welch’s *t*-tests, with *p* < 0.05 considered statistically significant. Results are reported as mean ± standard deviation.

## 3. Results

When an electric current passes through a microwire, it encounters resistance from the wire’s material, generating heat, a phenomenon known as resistive heating or Joule heating [60] (Equation (5)). The generated heat (*Q*) is proportional to the microwire’s resistance (R), the current (I), and the duration (t) for which the current flows.

(5) $$Q=RI^{2}t$$

For a microwire with a specific heat capacity and resistance, the temperature rise depends on I, t, and the rate of heat dissipation to the surroundings. In our experiments, we maintained a low current range, ensuring that the measured wire temperature reached a steady state within approximately 1 min, thereby stabilizing the wire’s surface temperature at each applied current. By controlling the current, we effectively regulated the temperature of the microwire.

### 3.1. Effect of Current on Electrothermal Polymerization

The polymerization of IPs is highly temperature-dependent. In our experiments, since the microwire’s surface temperature was regulated by the applied current, we hypothesized that varying the current could be used to control the thickness and uniformity of the CIP coating. Electrothermal polymerization was initiated by applying current through the microwire while it was immersed in the prepolymer solution within the chamber. Three different currents of 1.5 A, 2 A, and 2.5 A were tested, resulting in steady surface temperatures of 50 ± 4 °C (*n* = 3), 65 ± 5 °C (*n* = 3), and 85 ± 6 °C (*n* = 3), respectively.

**Figure 3:**
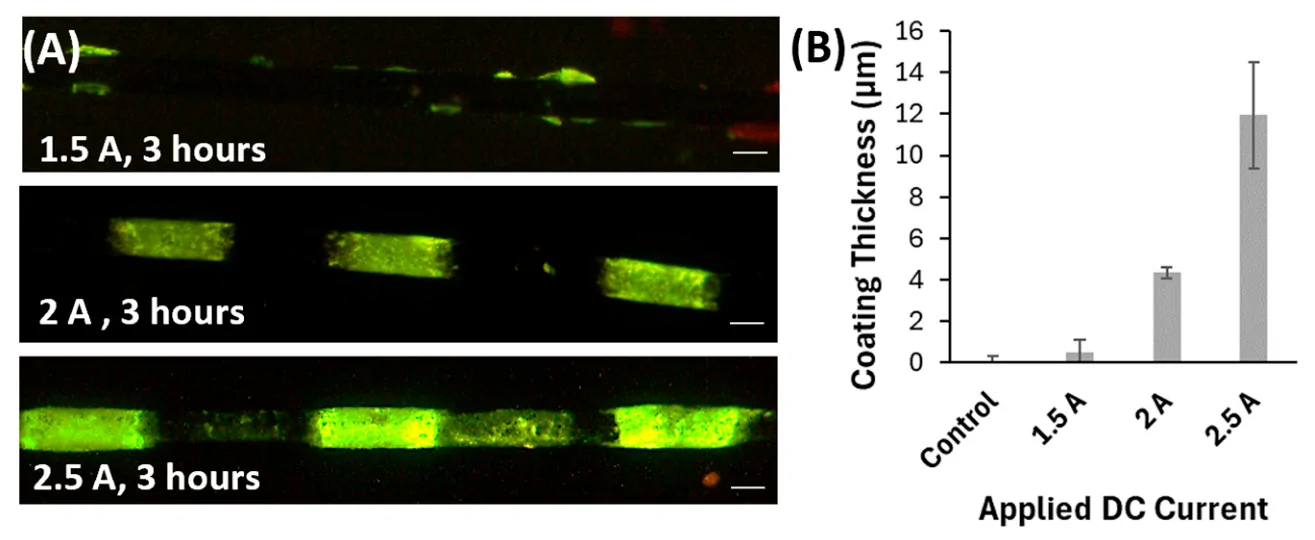
Effect of applied DC current on NIP coating of microwires in a single 3 h exposure step, followed by post-curing in the oven and washing. (**A**) Fluorescent images of microwires coated with DC currents of 1.5 A, 2 A, and 2.5 A applied for 3 h. (**B**) The thickness of coating islands shows a positive correlation with the applied current. Scale bars are 125 µm. Error bars are the standard deviation.

Figure 3 shows the effect of current on coating thickness. The average radius of bare wire was 64.3 ± 0.3 µm (*n* = 3), and coating thickness was determined by subtracting this value from the radius of the coated microwire.

Applying a DC current of 1.5 A for 3 h produced patchy coatings with sporadic spots on the microwire’s surface (Figure 3A). Increasing the current to 2 A and 2.5 A for the same duration resulted in discrete coating islands. Coating thickness measurements revealed a positive correlation between the applied current and coating thickness (Figure 3B): 1.5 A yielded 0.5 ± 0.6 µm (*n* = 3), 2 A produced 4.3 ± 0.3 µm (*n* = 3), and 2.5 A resulted in 12 ± 2.6 µm (*n* = 3). Similar coating patterns have been previously observed under specific polymer composition and polymerization conditions (temperature and time), using a conventional bulk polymerization approach [45].

To enhance coating uniformity and continuity along the microwires, a two-step electrothermal process was introduced. This method involved applying current for 3 h, briefly removing the microwire from the prepolymer solution for a few minutes and returning it back into the prepolymer solution, and then reapplying current for another 3 h (a total of 3 + 3 = 6 h current application). While this approach does not explain the underlying mechanism behind the formation of discrete coating islands, it significantly reduced their occurrence and improved the coating quality.

**Figure 4:**
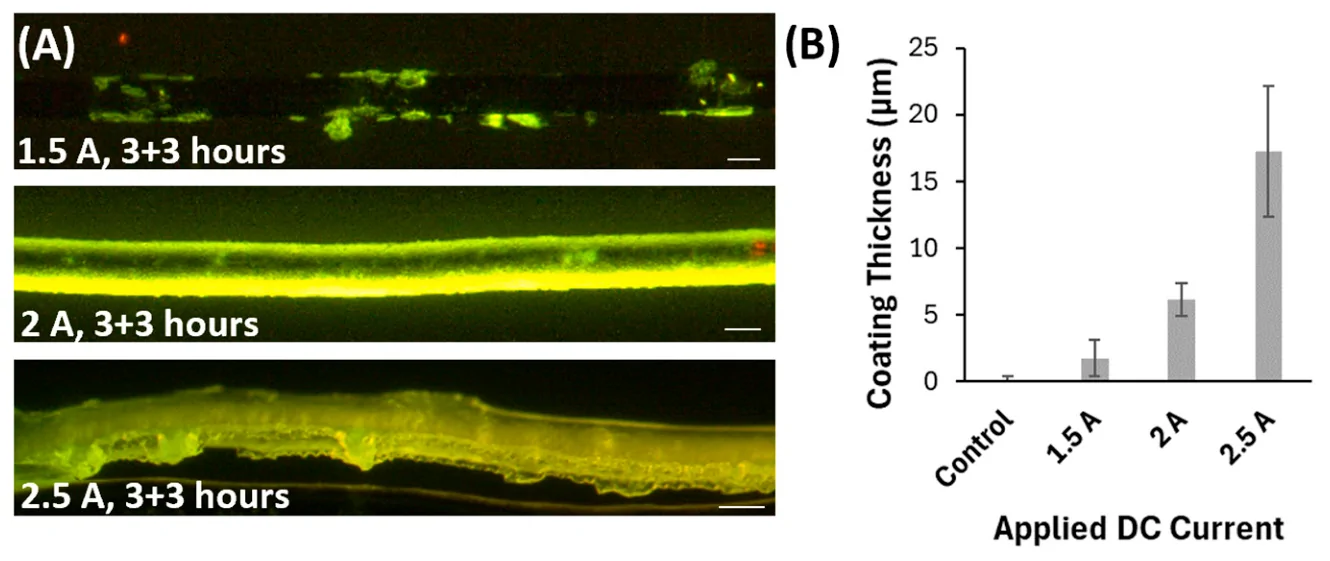
Effect of applied DC current on NIP coating of microwires in a two-step (3 + 3 h) exposure, followed by post-curing in the oven and washing. (**A**) Fluorescent images of microwires coated with DC currents of 1.5 A, 2 A, and 2.5 A applied for 3 + 3 h. (**B**) The coating thickness shows a positive correlation with the applied current. Scale bars are 125 µm. Error bars are the standard deviation.

A two-step electrothermal exposure (3 + 3 h) with a DC current of 1.5 A resulted in patchy coatings appearing as sporadic spots on the microwires’ surface (Figure 4A), similar to those observed with a single-step 3 h exposure with the same current. Increasing the current amplitude significantly increased the coating thickness, from 1.74 ± 1.3 µm (*n* = 3) at 1.5 A to 17.2 ± 4.9 µm (*n* = 3) at 2.5 A (Figure 4B). However, at 2.5 A, the coating became excessively thick and less uniform. An optimal current of 2 A for 3 + 3 h produced a uniform, continuous coating of 6.1 ± 1.2 µm (*n* = 3), suitable for bacterial imprinting and used throughout the experiments (Figure 4). The 2 A, 3 h condition, which produced discrete coating islands, resulted in an average *longitudinal coverage* of 50.09 ± 5.27% (*n* = 3). In contrast, microwires fabricated under the 2 A, 3 + 3 h condition exhibited continuous coating over the entire analyzed region, corresponding to a *longitudinal coverage* of 100% (*n* = 3) and an average uniformity index of 0.980±0.003 (n=3). These results quantitatively confirm that the two-step electrothermal exposure enhanced coating coverage, reduced diameter variation, and improved coating uniformity. At an applied current of 2.00 A, the output voltage displayed by the DC power supply was 0.41 ± 0.01 V, corresponding to an effective circuit resistance of 0.205 ± 0.005 Ω and an electrical power of approximately 0.82 ± 0.02 W. Because the voltage was not measured directly across the microwire segment, these values represent the effective electrical characteristics of the experimental circuit and may include contributions from the electrical contacts and connecting leads. Therefore, the resistance and power dissipated specifically by the microwire could not be independently determined.

### 3.2. Morphological Characterization of NIP- and CIP-Coated Microwires

**Figure 5:**
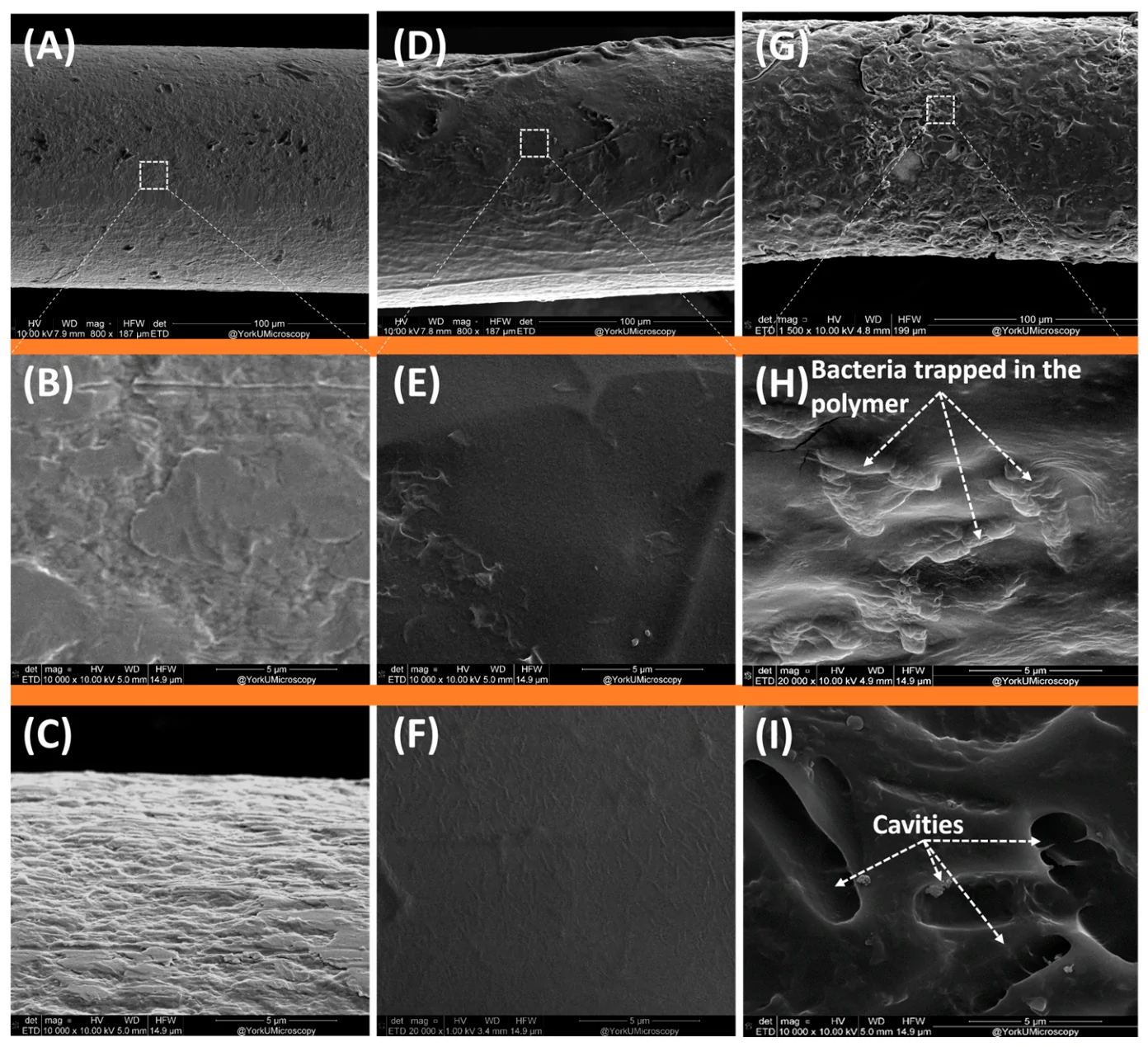
SEM images of bare and coated microwires. (**A**–**C**) Bare microwire, (**D**–**F**) microwires coated with non-imprinted polymer (NIP), and (**G**–**I**) microwires coated with cell-imprinted polymer (CIP). Higher magnification views of the bare microwire, NIP-coated microwire, and CIP-coated microwire before template washing are shown in (**B**), (**E**), and (**H**), respectively. The bare and NIP-coated microwires show no bacterial presence, while bacterial cells are visibly embedded in the polymer matrix of the CIP-coated microwire (**H**). The bare, NIP-coated and CIP-coated microwires after template washing are shown in (**C**), (**F**) and (**I**), respectively. Bacterial cavities are visible in (**I**).

SEM images in Figure 5 provide detailed insights into the morphology of bare microwires, NIP-coated microwires, and CIP-coated microwires at various experimental stages, both before (first and second rows) and after (third row) template washing. The bare microwires (Figure 5A–C) exhibited smooth surfaces without additional features, serving as an unmodified control. NIP-coated microwires (Figure 5D–F) showed uniform polymer deposition but lacked bacterial templates, confirming their role as non-imprinted controls.

In contrast, CIP-coated microwires (Figure 5G–I) revealed bacterial cells embedded within the polymer matrix before template washing (Figure 5G,H), demonstrating the successful incorporation of bacterial templates.

Following template washing, the bare and NIP-coated microwires (Figure 5C,F) retained their smooth surfaces, whereas the CIP-coated microwires (Figure 5I) exhibited distinct cavities corresponding to the removed bacterial templates. These cavities are consistent with morphological imprinting and may contribute to the enhanced rebinding observed for CIP-coated microwires relative to the controls.

**Figure 6:**
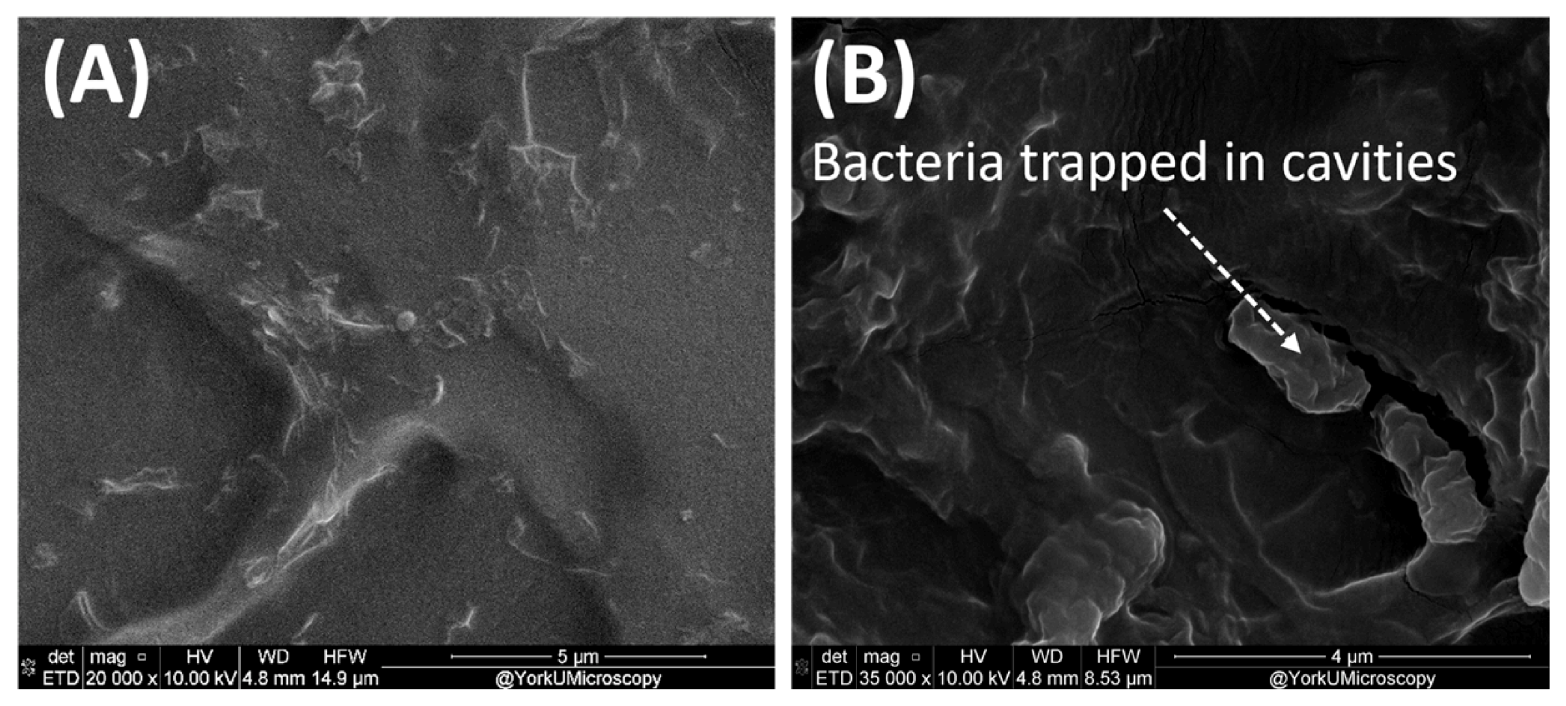
SEM images of NIP- and CIP-coated microwires after rebinding assay. (**A**) NIP-coated microwire after exposure to bacteria solution; (**B**) CIP-coated microwire after exposure to bacteria solution.

Figure 6 presents representative SEM images of NIP-coated and CIP-coated microwires following rebinding assays. Compared to the NIP-coated microwires, the CIP-coated microwires exhibited a greater number of bacterial cells attached to the polymer surface, with several cells appearing to occupy imprinted cavities. Although some bacterial attachment was also observed on the NIP-coated microwires, the higher bacterial retention on the CIP-coated microwires is consistent with the presence of recognition sites generated during the imprinting process. These observations, together with the quantitative rebinding results, support the enhanced bacterial capture capability of the CIP coatings while suggesting that non-specific interactions also contribute to binding.

### 3.3. Quantitative Evaluation of Bacterial Capture Performance

**Figure 7:**
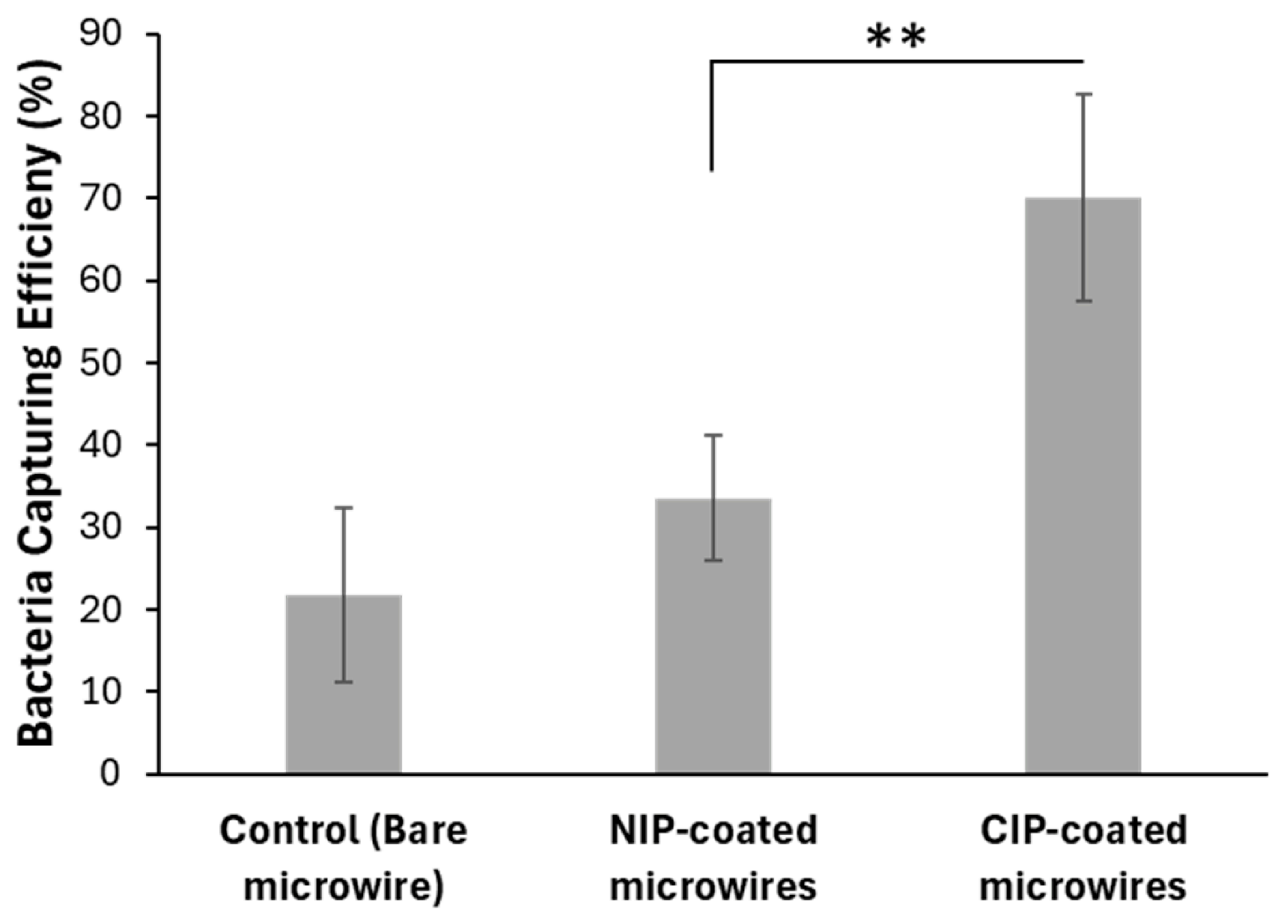
Bacterial capture performance of bare, NIP-coated, and CIP-coated microwires, evaluated by their ability to remove bacteria from solutions containing 10^4^ CFU/mL of *Salmonella*. Error bars represent standard deviations (*SD*s), and ** indicates a *p*-value < 0.01.

The *Salmonella* bacterial capture efficiencies of the CIP-coated microwires were evaluated using a rebinding assay and compared to those of bare and NIP-coated microwires. All experiments were performed at a bacterial concentration of ~10^4^ CFU/mL in quadruplicate (Table S3). As shown in Figure 7, both bare and NIP-coated microwires exhibited capture efficiencies of approximately 22% and 33%, respectively, with no statistically significant difference (*p* > 0.05, two-tailed Welch’s *t*-test). The slightly higher capture efficiency of the NIP-coated microwires compared to bare microwires may be due to favorable interactions between the bacteria and the NIP coating. In contrast, CIP-coated microwires achieved significantly higher bacterial capture efficiency of 70% compared to bare and NIP-coated microwires (*p* < 0.01, two-tailed Welch’s *t*-test). This substantial improvement supports the effectiveness of bacteria imprinting, which enables the CIP-coated microwires to capture bacteria within the imprinted cavities.

The initial concentration of ~10^4^ CFU/mL was selected to maintain consistency with previously reported rebinding protocols [45] and to provide sufficient colony counts for reliable quantification using the serial dilution and colony-counting method. In our previous study, post-rebinding samples prepared at lower concentrations, particularly below 10^2^ CFU/mL, frequently yielded colony numbers that were too low for robust enumeration. Because rebinding efficiency may vary with the initial bacterial concentration, the present results do not establish the concentration-dependent operating range of the CIP-coated microwires. Therefore, the present results should be interpreted as a proof-of-concept comparison of bare, NIP-coated, and CIP-coated microwires at a single bacterial concentration, rather than as a determination of the concentration-dependent operating range or detection limit. Future studies should evaluate rebinding performance across a broader concentration range, including lower bacterial loads and relevant biological or environmental sample matrices.

## 4. Discussion

Traditional approaches for immobilizing imprinted polymers (IP) on sensor transducers can fall into two main categories: (1) immobilizing pre-made IP particles via physical entrapment or chemical coupling and (2) directly preparing imprinted polymer films on transducer surfaces [61,62]. The method presented in this study aligns with the second category and can be compared to in situ self-assembly methods, including bulk [17], suspension [47], precipitation [48], and electrochemical [49] polymerization. These polymerization methods have limitations in achieving uniform coatings on complex geometries, particularly non-planar surfaces. For instance, bulk and suspension polymerization often tend to produce polymerization far from the transducer surface, leading to poor control over coating thickness and uniformity. Akhtarian et al. [45] used bulk polymerization to create CIP coatings on stainless steel microwires. Their CIP composition produced powder-like polymers after thermal polymerization, enabling the removal of microwires from the surrounding polymer matrix. However, for compositions that form harder, glass-like polymers—such as those used in this study—removing excess material and achieving precise thickness control with traditional methods become highly challenging. Electrochemical polymerization offers improved spatial control but is restricted to electrochemically compatible monomers and cross-linkers and typically requires planar electrodes, specialized instrumentation, and electrochemical expertise [63,64,65].

This study demonstrates that electrothermal polymerization, achieved by applying a DC current to a conductive substrate immersed in a prepolymer solution, provides a simple and effective method for fabricating cell-imprinted polymer (CIP) coatings on non-planar metallic microwires. By exploiting localized Joule heating, polymerization is confined to the microwire surface, enabling uniform coatings with controlled thickness while minimizing undesired bulk polymerization. The resulting CIP-coated microwires exhibited significantly higher *Salmonella* capture efficiencies than bare and non-imprinted polymer (NIP)-coated controls, demonstrating that electrothermal polymerization can successfully generate functional recognition sites for bacterial capture. The precise control of polymer thickness and uniformity is particularly important for sensor performance, as these properties influence analyte diffusion, the accessibility of recognition sites, and overall sensitivity. While planar transducers such as quartz crystal microbalances and screen-printed electrodes facilitate thickness control via deposition techniques, extending the same level of control to non-planar substrates remains challenging. Metallic microwires offer several attractive features for sensing applications, including high surface-to-volume ratios, low cost, and straightforward integration into microfluidic platforms [66,67,68,69]. The electrothermal coating strategy developed here enables localized coatings on these geometries while allowing coating thickness to be tuned through the applied current and polymerization time.

Currently, the present work is limited to a proof-of-concept evaluation using a single bacterial species and a single initial concentration in the rebinding assay. Selectivity against non-target or mixed bacterial populations, concentration-dependent capture performance, and the practical operating range of the coated microwires were not assessed and should be investigated in the future. Template-removal efficiency and possible HCl-induced changes to the polymer network should also be examined in future studies. Additional work will also be required to integrate the coated microwires with an appropriate signal-transduction method and to evaluate their performance in operational sensing or microfluidic devices. The chamber design may permit more than one microwire to be coated during a single fabrication cycle. However, fabrication throughput, batch-to-batch reproducibility, maximum wire capacity, and scale-up were not evaluated in the present study. Moreover, future work may investigate the effects of polymer composition and surface functionalization across different substrate materials and target analytes. The further optimization of these parameters will improve coating quality, reproducibility, and scalability, facilitating the broader application of electrothermal polymerization in biosensing, diagnostics, and selective analyte extraction.

## 5. Conclusions

This study demonstrates an electrothermal approach for fabricating imprinted polymer coatings directly on non-planar metallic microwires. Localized Joule heating of microwires immersed in the prepolymer solution enabled polymerization at the microwire surface while minimizing undesired polymerization within the bulk solution. The applied current and polymerization time were identified as key parameters influencing coating formation and were used to control coating thickness and continuity. The selected condition of 2 A applied for 3 + 3 h produced a continuous coating with a thickness of 6.1 ± 1.2 µm. SEM imaging showed bacterial templates embedded within the polymer before washing and bacteria-shaped cavities following template removal. Rebinding experiments further demonstrated enhanced *Salmonella* capture by CIP-coated microwires, with an approximately 70% capture efficiency compared with 33% for NIP-coated microwires and 22% for bare microwires.

The present study was limited to a single bacterial species and did not evaluate non-target or mixed-species samples. Future studies should evaluate concentration-dependent capture, cross-species selectivity, fabrication reproducibility, and the integration of the coated microwires into operational sensing platforms. The present findings establish the feasibility of electrothermal CIP fabrication and bacterial capture, providing a foundation for the future development and validation of integrated biosensing devices.

## Data Availability

Data are available upon reasonable requests to interested researchers.

## Supplementary Materials

The following supporting information can be downloaded at https://www.mdpi.com/article/10.3390/s26175324/s1: Note S1: Measurement of microwire diameter and coating coverage. Figure S1: Image-processing workflow for microwire diameter measurement: (A) original microscopic image; (B) grayscale image; (C) Canny edge-detection output [70]; and (D) inter-edge distance measurements at 100 randomly selected points. Table S1: Individual microwire diameter measurements. Wire diameters were measured after electrothermal polymerization under different applied currents and durations. Each reported diameter represents the mean of 100 inter-edge measurements obtained along an approximately 2 mm-long region of one independently prepared microwire. Table S2: Longitudinal coating coverage of NIP-coated microwires fabricated under 2 A, 3 h and 2 A, 3 + 3 h electrothermal polymerization conditions. Note S2: Plate culturing and colony counting. Table S3: Raw colony counts obtained from 100 µL aliquots of 10-fold diluted residual bacterial suspensions following rebinding with bare, NIP-coated, and CIP-coated microwires.

## Abbreviations

The following abbreviations are used in this manuscript:

AAM	Acrylamide
ABAH	2,2′-Azobis(2-amidinopropane) hydrochloride
AIBN	2,2′-Azobisisobutyronitrile
CFU	Colony-forming unit
CIP	Cell-imprinted polymer
CIP-MW	Cell-imprinted polymer-coated microwire
DC	Direct current
DHEBA	*N*,*N*′-(1,2-dihydroxyethylene) bisacrylamide
DI	Deionized
DMSO	Dimethyl sulfoxide
EDC	*N*-(3-Dimethylaminopropyl)-*N′*-ethylcarbodiimide hydrochloride
ELISA	Enzyme-linked immunosorbent assay
HCl	Hydrochloric acid
H_2_O_2_	Hydrogen peroxide
IIP	Ion-imprinted polymer
IP	Imprinted polymer
LB	Luria–Bertani
MAA	Methacrylic acid
MIP	Molecularly imprinted polymer
MMA	Methyl methacrylate
MUA	11-Mercaptoundecanoic acid
MW	Microwire
NHS	*N*-Hydroxysuccinimide
NIP	Non-imprinted polymer
NIP-MW	Non-imprinted polymer-coated microwire
NVP	*N*-Vinylpyrrolidone
PCR	Polymerase chain reaction
PDMS	Polydimethylsiloxane
QCM	Quartz crystal microbalance
SEM	Scanning electron microscopy
SPE	Screen-printed electrode
