# Supplementary material for: Electrothermal Synthesis of Cell-Imprinted Polymer Coatings on Metallic Microwires for Bacterial Capture
Source: Sensors (Basel). 2026 Aug 22;26(17):5324. doi: 10.3390/s26175324 (PMC13568193; doi:10.3390/s26175324)
Supplement: Supplementary file 1 [file sensors-26-05324-s001.zip › sensors-4437501-supplementary.pdf]

Supporting Information

Electrothermal Synthesis of Cell-Imprinted Polymer Coatings on Metallic Microwires for Bacterial Capture

Alireza Zabihihesari [1,2,†], Arezoo Khalili [1,†] and Pouya Rezai [1,3,*]

[1] Department of Mechanical Engineering, York University, Toronto, ON M3J 1P3, Canada; alireza.zabihihesari@dal.ca (A.Z.); arezookhalili1050@gmail.com (A.K.)
[2] Department of Electrical and Computer Engineering, Dalhousie University, Halifax, NS B3H 4R2, Canada
[3] Department of Mechanical Engineering, George Mason University, Fairfax, VA 22030, USA
* Correspondence: pouya.rezai@lassonde.yorku.ca
† These authors contributed equally to this work.

Note S1: Measurement of microwire diameter and coating coverage

A custom-developed macro in ImageJ software [1] was used to measure microwire diameters (Fig. S1). The microwire images were first converted to grayscale to increase contrast. Then, Canny edge detection function [2] was utilized to detect the edges of the microwires. When automatic edge detection was incomplete or inaccurate, the corresponding edge segments were manually traced in ImageJ before measurement. The macro randomly selected 100 points along one edge and calculated the shortest distance from each point to the opposite edge. The mean of inter-edge distance was reported as the microwire diameter along with standard deviations (Table S1).

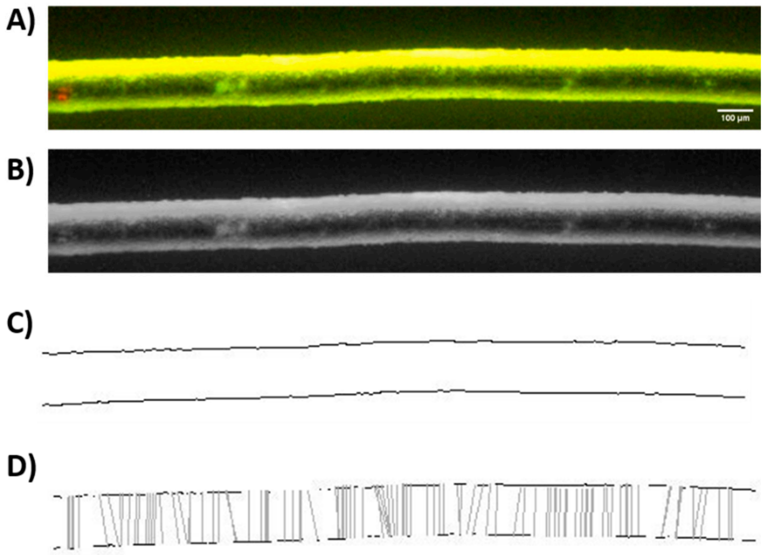

Figure S1. Image-processing workflow for microwire diameter measurement: (A) original microscopic image; **(B)** grayscale image; **(C)** Canny edge-detection output; and **(D)** inter-edge distance measurements at 100 randomly selected points.

Table S1: Individual microwire diameter measurements. Wire diameters were measured after electrothermal polymerization under different applied currents and durations. Each reported diameter represents the mean of 100 inter-edge measurements obtained along an approximately 2 mm-long region of one independently prepared microwire.

Sample	Applied current (A)	Current application time (h)	Measured microwire diameter (µm)	Measured standard deviation (µm)
Bare wire	0 (control)	-	128.00	0.89
Bare wire	0 (control)	-	128.27	0.78
Bare wire	0 (control)	-	129.30	1.10
NIP-coated	1.5	3	128.60	12.11
NIP-coated	1.5	3	129.00	10.75
NIP-coated	1.5	3	130.96	15.35
NIP-coated	2	3	136.64	8.70
NIP-coated	2	3	137.31	12.29
NIP-coated	2	3	137.65	11.78
NIP-coated	2.5	3	149.00	10.30
NIP-coated	2.5	3	150.00	10.51
NIP-coated	2.5	3	158.30	11.10
NIP-coated	1.5	3+3	133.00	17.10
NIP-coated	1.5	3+3	134.02	15.20
NIP-coated	1.5	3+3	129.10	14.50
NIP-coated	2	3+3	140.00	2.65
NIP-coated	2	3+3	138.90	3.41
NIP-coated	2	3+3	143.50	2.65
NIP-coated	2.5	3+3	174.00	11.91
NIP-coated	2.5	3+3	160.01	12.02
NIP-coated	2.5	3+3	155.00	9.74

Table S2. Longitudinal coating coverage of NIP-coated microwires fabricated under 2 A, 3 h and 2 A, 3+3 h electrothermal polymerization conditions.

Condition	Total length analyzed (µm)	Individual coated segments (µm)	Total coated length (µm)	Longitudinal coverage (%)
2 A, 3 h	2087.94	518.77, 537.36	1056.13	50.58
2 A, 3 h	2190.50	408.64, 380.70, 417.61	1206.95	55.10
2 A, 3 h	2552.16	373.69, 398.46, 366.02	1138.17	44.60
2 A, 3+3 h	1950.87	Continuous coating	Equal to analysed length	100
2 A, 3+3 h	2025.30	Continuous coating	Equal to analysed length	100
2 A, 3+3 h	2110.50	Continuous coating	Equal to analysed length	100

Note S2: Plate culturing and colony counting

Bacterial concentrations before and after the rebinding assay were determined by plate culturing and colony counting. Aliquots of the initial bacterial suspension, and the residual suspension obtained after the 30 min rebinding period and removal of the microwire were diluted 10-fold. A 100 µL aliquot of each diluted suspension was spread onto LB agar plates and incubated at 37 °C for 20 h, after which colonies were counted.

Bacterial concentration in CFU/mL was calculated as:

$$B = \frac{N \times DF}{V} \qquad \text{(S1)}$$

where N is the number of colonies counted, DF is the dilution factor (10), and V is the plated volume (0.1 mL). Thus, under the conditions used here:

$$B = N \times 100 \quad \text{(CFU/mL)} \qquad \text{(S2)}$$

The average initial colony count was 106 colonies, corresponding to an initial bacterial concentration of 1.06×10^4 CFU/mL. Raw residual colony counts from four independent rebinding replicates for each microwire condition are presented in Table S3 and were used to calculate bacterial capture efficiency.

Table S3. Raw colony counts obtained from 100 µL aliquots of 10-fold diluted residual bacterial suspensions following rebinding with bare, NIP-coated, and CIP-coated microwires.

Replicate	Bare microwire	NIP-coated microwire	CIP-coated microwire
1	89	75	50
2	95	59	32
3	78	71	26
4	70	77	19

References

1. Akhtarian, S.; Doostmohammadi, A.; Youssef, K.; Kraft, G.; Kaur Brar, S.; Rezai, P. Metal Microwires Functionalized with Cell-Imprinted Polymer for Capturing Bacteria in Water. *ACS Appl. Polym. Mater.* **2023**, *5*, 3235–3246.

2. Canny, J. A Computational Approach to Edge Detection. *IEEE Trans. Pattern Anal. Mach. Intell.* **1986**, *PAMI-8*, 679–698, doi:10.1109/TPAMI.1986.4767851.
